# Prohibition of antibiotic growth promoters has affected the genomic profiles of *Lactobacillus salivarius* inhabiting the swine intestine

**DOI:** 10.1371/journal.pone.0186671

**Published:** 2017-10-23

**Authors:** Jun-Yeong Lee, Geon Goo Han, Ho-Bin Lee, Sang-Mok Lee, Sang-Kee Kang, Gwi-Deuk Jin, Jongbin Park, Byung Jo Chae, Yo Han Choi, Eun Bae Kim, Yun-Jaie Choi

**Affiliations:** 1 Department of Agricultural Biotechnology, Seoul National University, Seoul, Republic of Korea; 2 Institute of Green-Bio Science & Technology, Seoul National University, Pyeongchang, Republic of Korea; 3 Department of Animal Life Science, Kangwon National University, Chuncheon, Republic of Korea; 4 Research Institute for Agriculture and Life Science, Seoul National University, Seoul, Republic of Korea; Institut National de la Recherche Agronomique, FRANCE

## Abstract

After the introduction of a ban on the use of antibiotic growth promoters (AGPs) for livestock, the feeding environment, including the composition of animal intestinal microbiota, has changed rapidly. We hypothesized that the microbial genomes have also been affected by this legal prohibition, and investigated an important member of the swine gut microbiota, *Lactobacillus salivarius*, with a pan-genomic approach. Here, we isolated 21 *L*. *salivarius* strains composed of 6 strains isolated before the AGP prohibition (SBPs) and 15 strains isolated after the AGP prohibition (SAPs) at an interval of a decade, and the draft genomes were generated *de novo*. Several genomic differences between SBPs and SAPs were identified, although the number and function of antibiotic resistance genes were not different. SBPs showed larger genome size and a higher number of orthologs, as well as lower genetic diversity, than SAPs. SBPs had genes associated with the utilization of L-rhamnose and D-tagatose for energy production. Because these sugars are also used in exopolysaccharide (EPS) synthesis, we tried to identify differences in biofilm formation-associated genes. The genes for the production of EPSs and extracellular proteins were different in terms of amino acid sequences. Indeed, SAPs formed dense biofilm and survived better than SBPs in the swine intestinal environment. These results suggest that SAPs have evolved and adapted to protect themselves from new selection pressure of the swine intestinal microenvironment by forming dense biofilms, adopting a distinct antibiotic resistance strategy. This finding is particularly important to understand the evolutionary changes in host-microbe interaction and provide detailed insight for the development of effective probiotics for livestock.

## Introduction

Antibiotic growth promoters (AGPs) have been added to livestock diets since the 1950s [1]. They have provided increased productivity to farmers, and the agricultural industries and the consumers have benefited from them. In the last few decades, scientists and politicians have expressed concerns about the use of antibiotics in animal feed. These include the potential development and transfer of antibiotic resistance of bacteria, which could become resilient to medicines used to treat humans, leading to increased illnesses and mortality in humans [2]. An action supporting the ban of AGPs from livestock feed was started in Europe. Sweden was the first country to ban the use of all AGPs, and since then many countries have prohibited the addition of AGPs to animal feeds [3]. Republic of Korea also adopted this legislation, and the regulations came into force on July 2011 [4].

Several studies have suggested that a persistent treatment with antibiotics, such as AGPs in feeds, has an impact on the intestinal bacteria of animals and their habitats [5–7]. Moreover, numerous reports have revealed that the use of antibiotics leads to the evolution of bacteria [8–10]. Changes in bacterial strategies for overcoming antibiotic stress occur rapidly and extensively through genetic mutations [11]. In contrast, studies on the evolutionary evidence of bacteria after the ban of AGPs have been limited to the analysis of decreased antibiotic resistance of commensal bacteria [12, 13].

We corroborated the influence of AGP prohibition on *Lactobacillus salivarius* with a focus on the evolutionary aspect. *L*. *salivarius* is a Gram-positive and lactic acid-producing bacterium and is an important member of the swine intestinal microbiota [14]. This species is a promising probiotic candidate frequently isolated from human, porcine and avian gastrointestinal tracts, and the strains are bacteriocin producers [15]. 21 *L*. *salivarius* strains had been isolated from several swine farms before and after the ban of AGPs at an interval of a decade, and we used previously generated draft genomes for pan-genomic analysis [16]. This approach provided a valuable insight into genomic variation, including niche specificity, evolution of species, antibiotic resistance and many other features of *L*. *salivarius*.

## Results

### Preparation of *L*. *salivarius* genomes

We isolated the *L*. *salivarius* strains that inhabited the intestinal tracts of swine from several farms of Republic of Korea under two different conditions: 1) AGPs had been used as feed additives for livestock (in 2005 and 2006), and 2) after the ban on AGP addition to the feeds (in 2014 and 2015). *L*. *salivarius* strains composed of 89 strains from before AGP prohibition and 195 strains from after AGP prohibition were isolated and identified as described in the previous study [16]. Among them, 21 *L*. *salivarius* strains including six strains isolated before AGP prohibition (SBPs) and fifteen strains isolated after AGP prohibition (SAPs) were randomly selected.

Whole genome sequencing of 6 SBPs and 15 SAPs had been carried out, and draft genomes of the strains had been generated as described in the previous study [16] (Table 1). Average nucleotide identity based on BLAST+ (ANIb) values were calculated to confirm the taxa of these strains. All of the strains showed over 97% of ANIb values through pairwise comparison with the reference genome of the species, *L*. *salivarius* UCC118, indicating that all of the strains used in this study belonged to the same species, *L*. *salivarius* [17].

**Table 1:** Draft genomes used in this study.

Group	Strain	Sampling farm	Genome size (Mbp)	No. of CDSs	GC %	NCBI accession
SBP	KLA001	A	2.27	2171	32.83	LXZT00000000
SBP	KLA005	A	2.26	2177	32.82	LXZP00000000
SBP	KLA002	B	2.26	2175	32.82	LXZS00000000
SBP	KLA003	C	2.27	2169	32.83	LXZR00000000
SBP	KLA004	D	2.26	2180	32.83	LXZQ00000000
SBP	KLA006	E	2.37	2265	32.93	LXZO00000000
SAP	KLF002	G	2.18	2098	32.81	LXZM00000000
SAP	KLF003	G	2.21	2128	32.73	LXZL00000000
SAP	KLF004	G	2.12	2036	32.71	LXZK00000000
SAP	KLF005	F	2.15	2020	32.69	LXZJ00000000
SAP	KLF007	F	2.22	2138	32.9	LXZH00000000
SAP	KLW005	H	2.33	1971	32.82	LXZC00000000
SAP	KLW001	H	2.09	2194	33.04	LXZG00000000
SAP	KLW006	F	2.37	1892	32.7	LXZB00000000
SAP	KLW007	F	2.39	1819	32.75	LXZA00000000
SAP	KLW003	H	2.08	2233	32.92	LXZE00000000
SAP	KLW008	F	2.03	2011	32.88	LXYZ00000000
SAP	KLW009	F	1.97	1961	32.86	LXYY00000000
SAP	KLW010	F	2.14	2148	32.67	LXYX00000000
SAP	KLW004	H	2.07	2185	32.82	LXZD00000000
SAP	KLW002	H	2.39	1982	32.82	LXZF00000000

The 21 genomes obtained in this study had 3,318 total orthologous coding DNA sequences (CDSs), including 1,384 core genes and 322 stain-specific orthologs (S1 Fig). The number of orthologs only appeared in SBPs (335 orthologs) and was higher than the number of SAP-specific orthologs (103 orthologs). As shown in Table 2, the average genome size of SBPs was significantly larger than that of SAPs, and SBPs had a higher number of CDSs than SAPs. GC% was not different between the two groups.

**Table 2:** Averages of genomic features in SBPs and SAPs (mean ± standard deviation).

Group	Genome size (Mbp)	No. of CDSs	GC %
SBPs	2.28 ± 0.04	2189.50 ± 37.20	32.84 ± 0.04
SAPs	2.18 ± 0.13	2054.40 ± 118.81	32.81 ± 0.10
P-value	0.01	0.0009	0.27

**Fig 1:**
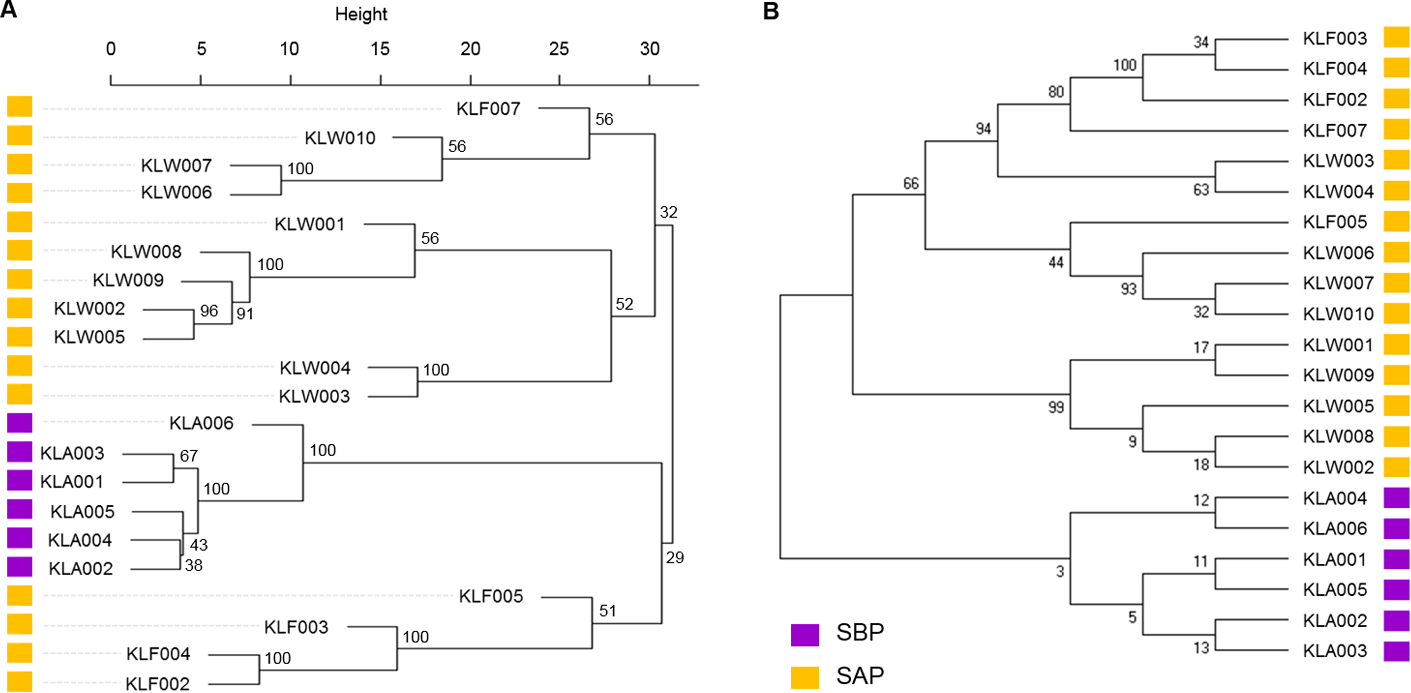
Phylogenetic tree of the isolated *L*. *salivarius* strains. (A) Hierarchical clustering of *L*. *salivarius* strains based on orthologous CDS contents. (B) Phylogenetic analysis of MLST sequences found in the strains. The bootstrap consensus tree inferred from 1,000 replicates is taken.

To confirm whether SBPs and SAPs are different in the genomic contents, hierarchical clustering of the genomes was carried out. The clustering based on 3,318 orthologous CDSs showed that the 6 SBPs were distinguished from 15 SAPs (Fig 1A). Another phylogenetic analysis based on the sequences of seven multilocus sequence typing (MLST) genes also showed that SBPs are strictly separated from SAPs (Fig 1B). Considering these distinctions between the two groups, it is evident that SBPs formed an indisputably distinct lineage from SAPs, and AGPs in feeds affect the genomic contents of *L*. *salivarius* that colonized the porcine intestine. Furthermore, the genetic distance between SBPs was closer than SAPs and this indicated that SBPs had more similar genomic features, although SBPs were isolated from more variable swine farms than SAPs.

### Antibiotic resistance of SAPs and SBPs

**Fig 2:**
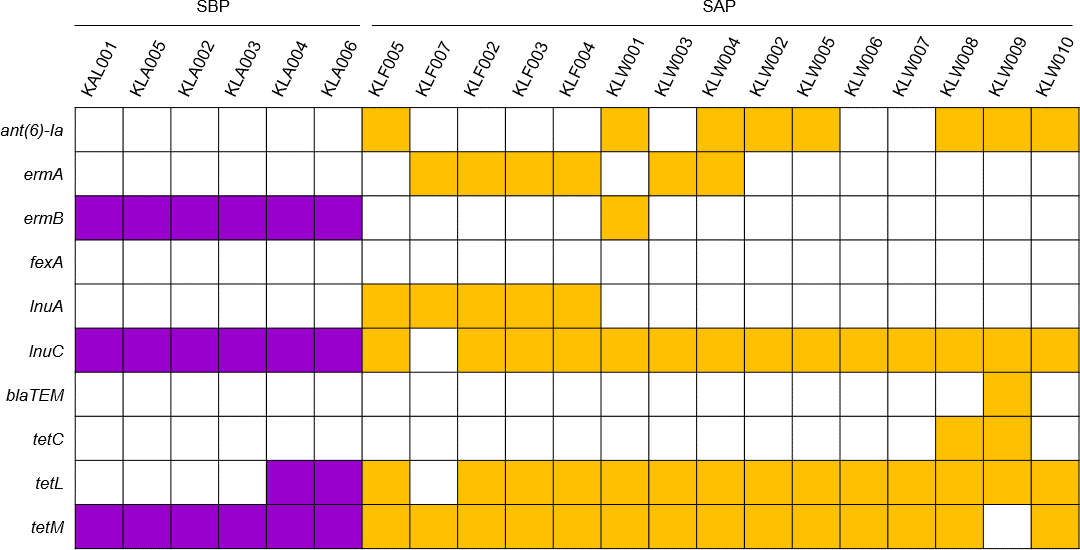
Comparison of AR genes of the isolated strains. Nucleotide sequences of AR genes were obtained from CARD. Orthologs of *L*. *salivarius* strains were aligned to the database using BLAST+, and orthologs showing E-value = 0 and identity ≥ 90% were regarded as AR genes (colored square).

As described above, the isolation conditions of SBPs and SAPs were different. Bacteria that had colonized the animal intestines had acquired antibiotic resistance (AR) genes for their adaptation to the environment in which farmers had used AGPs. We confirmed the presence of AR genes in the genomes using a database of AR genes, the Comprehensive Antibiotic Resistance Database (CARD) (Fig 2). All of the strains had three to five AR genes and there was no difference in the number of AR genes existing in the genomes, although it is no longer necessary for SAPs to maintain the AR genes after the use of AGPs was banned. The ancestors of SAPs acquired AR genes to protect themselves against antibiotics when AGPs were used freely, and SAPs have maintained these AR genes inherited from their ancestors until recently.

The different distribution of AR genes in SBPs and SAPs might be influenced by isolated farms, but these genes were analogous in their functions. The AR genes target the same antibiotics class, protein synthesis inhibitors, except the *blaTEM* gene, which protects bacteria from penicillins, one of the cell envelope antibiotics, indicating that functionally similar or identical AGPs had been used in the swine farms of Republic of Korea. Most of these AR genes are act by blocking the protein synthesis inhibitors to protect *L*. *salivarius* strains from lincosamides (*ermA*, *ermB*, *lnuA*, *lnuC* and *vatE*) and tetracyclines (*tetC*, *tetL* and *tetM*). Other two AR genes, *ant(6)-Ia* and *fexA*, have a defensive function against aminoglycosides and amphenicols, respectively.

### Functional differences in genomic contents

**Fig 3:**
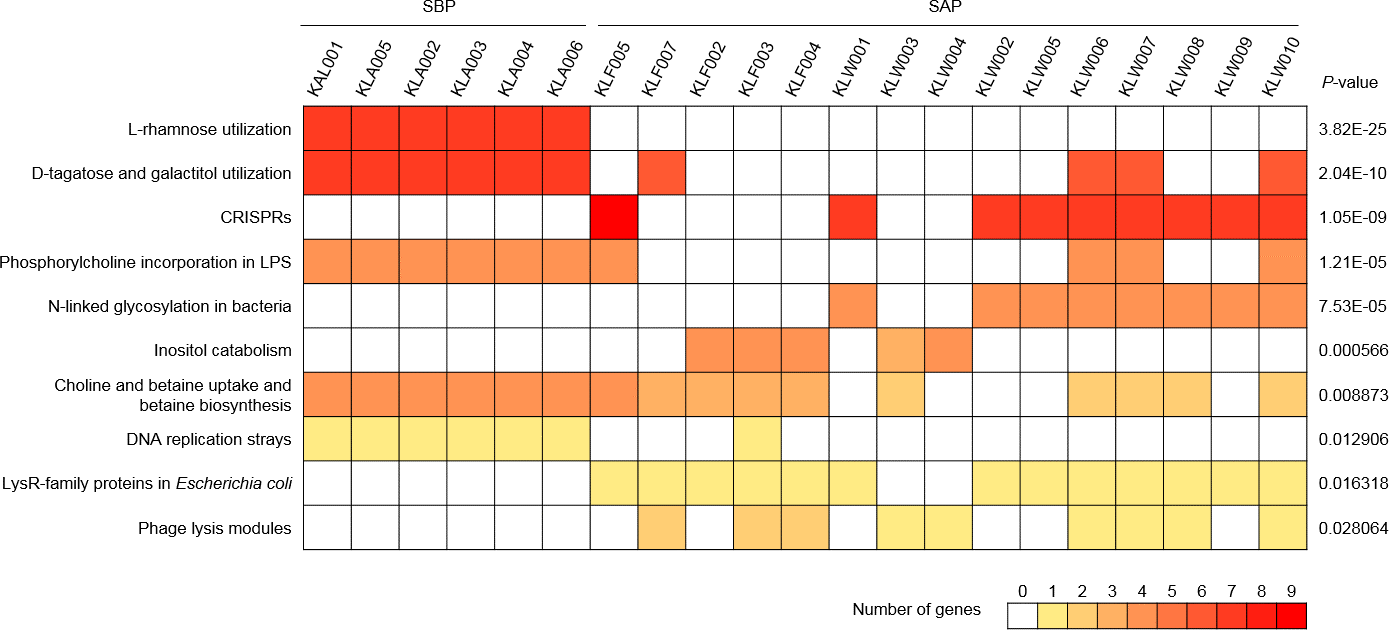
Comparison of RAST subsystems of the isolated genomes. The genomes were annotated and grouped by RAST with default options for bacteria. The different colors show the number of genes that incorporated in each subsystem (see color bar). Statistical analysis was carried out using Fisher's exact test between SBPs and SAPs, and the significantly different subsystems are shown in the figure (P < 0.05).

Genomic annotation using the Rapid Annotation using the Subsystem Technology (RAST) server revealed the genomic differences between SBPs and SAPs. We found 10 RAST subsystems that are significantly different between the groups (P < 0.05, Fig 3). Among these subsystems, L-rhamnose utilization and D-tagatose and galactitol utilization of SBPs had over seven genes per strain for each subsystem, and these genes were almost absent in the genome of SAPs. These two subsystems are responsible for energy metabolism. Thus, SBPs utilize L-rhamnose, D-tagatose and galactitol as carbon sources for energy production. Genes for L-rhamnose utilization were incorporated in glycolysis and pyruvate metabolism (Fructose and mannose metabolism, KEGG pathway ec00051), and genes for D-tagatose and galactitol utilization were related to the pentose phosphate pathway (Galactose metabolism, KEGG pathway ec00052). The other eight subsystems showed significant differences between groups, but these subsystems sparsely existed in the 21 strains (Fig 3).

### Comparative genomics focusing on genes for the production of cell wall components

L-rhamnose and D-tagatose are not only incorporated in the energy production pathways, but are also important for exopolysaccharide (EPS) production [18–20]. We postulated that SBPs would rather use L-rhamnose and D-tagatose as energy sources than for EPS production, due to the presence of the genes for L-rhamnose and D-tagatose utilization considering their associated metabolic mechanisms. If SBPs used these sugars for energy production rather than EPS synthesis, their genomes would have changed to reflect the decreased use of L-rhamnose and D-tagatose for EPS synthesis.

**Fig 4:**
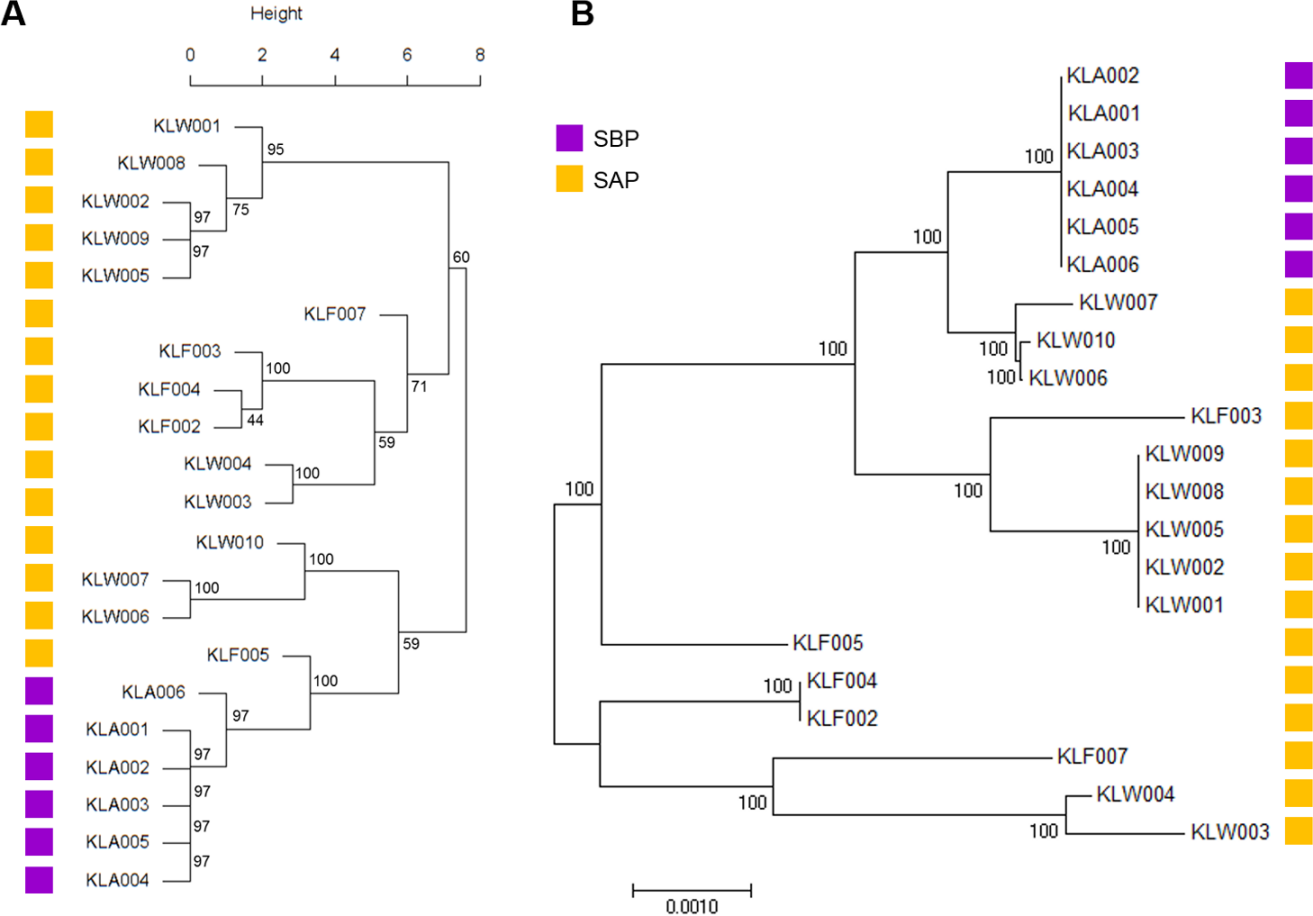
Phylogenetic clustering with EPS-related genes of SAPs and SBPs. (A) Tree based on the presence of 128 EPS-related genes of SAPs and SBPs. (B) Hierarchical clustering based on amino acid sequences of the 40 core genes for EPS production. The bootstrap consensus tree inferred from 1,000 replicates is taken.

Therefore, we investigated the absence or presence of the genes associated with EPS production. 217 genes related to EPS synthesis were collected by screening against Pfam, including 50 genes used in the previous study [21]. Among them, 128 orthologs including 40 core genes were found in the SBPs and SAPs. Gene presence in SBPs was different from the genomes of SAPs, furthermore, the phylogenetic diversity of the amino acid sequence of the genes with hierarchical clustering also revealed the distance between SBPs and SAPs (Fig 4). In Gram-positive bacteria, EPSs are important membrane components for niche adaptation and colonization through the biofilm formation [22], suggesting that the bacteria improved their ability for biofilm formation by the mutation and gene acquisition in EPS genes.

**Fig 5:**
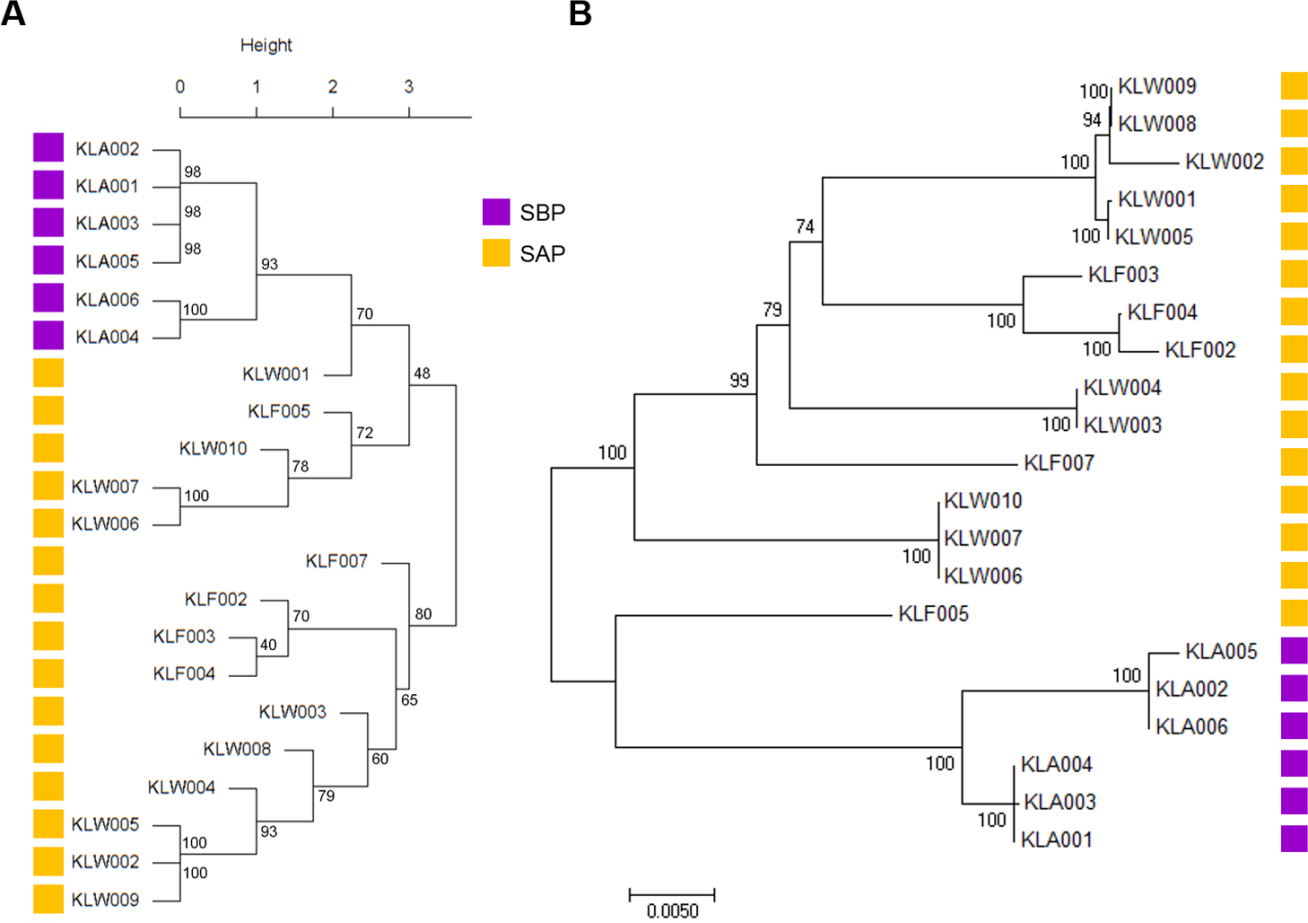
Phylogenetic clustering with extracellular protein genes of SAPs and SBPs. (A) Tree based on the presence of 30 extracellular protein genes of SAPs and SBPs. (B) Hierarchical clustering based on amino acid sequences of the 14 core genes for extracellular protein. The bootstrap consensus tree inferred from 1,000 replicates is taken.

The genetic profiles for the extracellular proteins which are also associated to biofilm formation were also determined. Among the 56 extracellular protein-encoding orthologs in the pan-genome, 30 orthologs were observed in the SBPs and SAPs, consisting with 7 choline-binding domains, 11 LPXTG domains (sortase-dependent proteins), one lipoprotein anchor, 10 LysM domains and one peptidoglycan-binding domain. Phylogenetic clustering showed that the ortholog distribution of SBPs was distinct from SAPs, implying the evolutionary differences between the groups (Fig 5A). Furthermore, hierarchical clustering based on amino acid sequences of the 14 core genes for extracellular proteins in the SBPs and SAPs also showed the genetic distance between them (Fig 5B).

The analyses of EPS and extracellular protein genes revealed that the amino acid sequence of the SBP genes was distinct from SAPs, suggesting that these differences would affect the biofilm formation of the two groups. We also analyzed copy number variation of the EPS genes and the extracellular protein genes found in the *L*. *salivarius* strains, but no significant differences were detected between SBPs and SAPs.

### *In vitro* biofilm formation and competition with intestinal microorganisms

**Fig 6:**
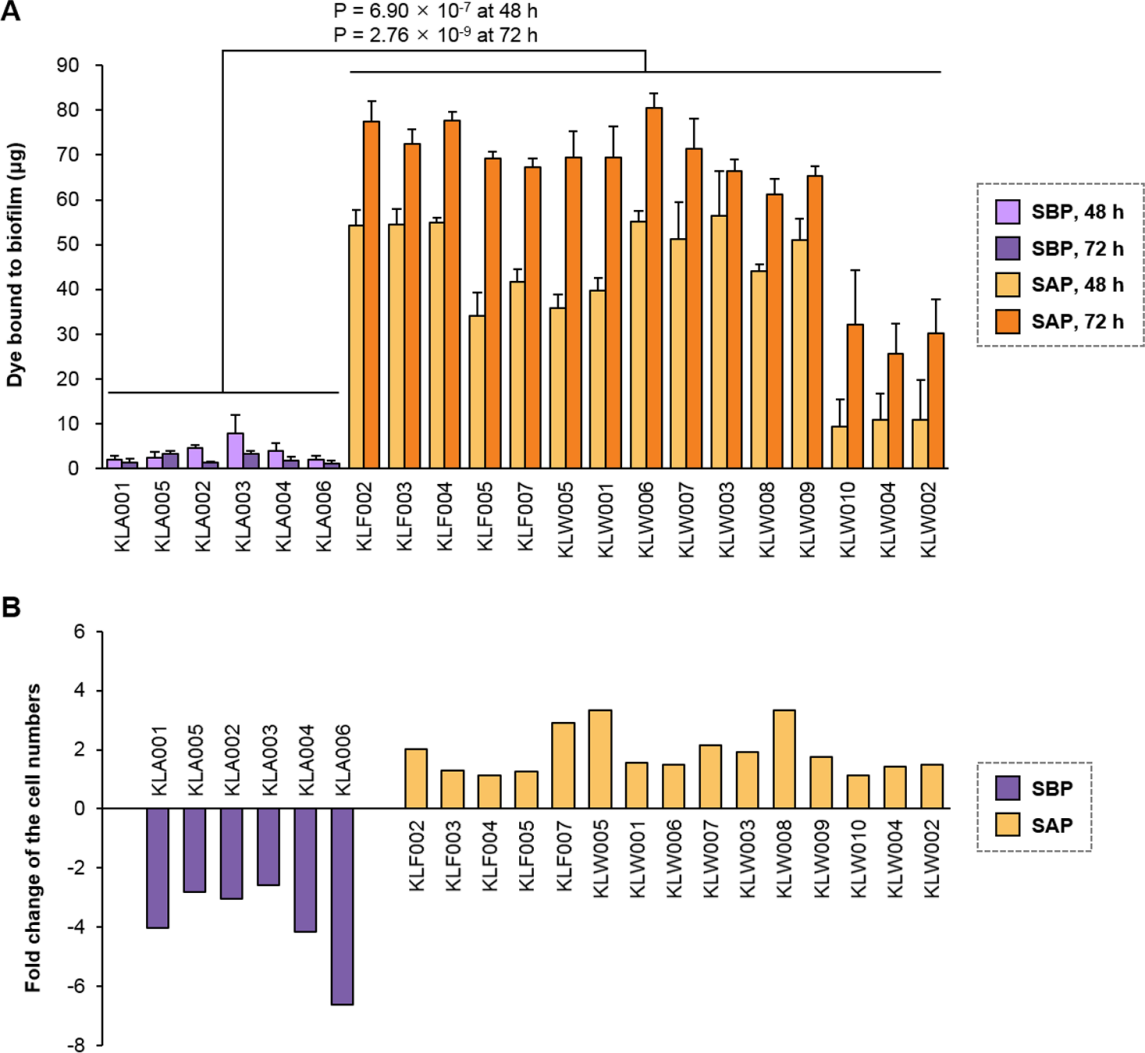
*In vitro* confirmation of the survival characteristics. (A) Detection of biofilm formation at 48 h and 72 h after incubation. (B) Fold change of the cell numbers of the isolated strains in competition with swine intestinal microbiota. The difference in the cell numbers between 24 h and 48 h after incubation was calculated. The cell number quantification results are shown in S3 Fig.

EPSs and extracellular proteins are major constituents of the Gram-positive bacterial cell wall and are important for biofilm formation of *L*. *salivarius* [21, 23]. Furthermore, different cell aggregation between SBPs and SAPs was observed (S2 Fig). Cell aggregation is also affected by cell wall components and associated with biofilm formation [24]. Considering that the distribution and peptide sequences of EPS and extracellular protein genes were different between SBPs and SAPs, it is assumed that ability for biofilm formation of the strains would be different. *In vitro* biofilm formation of the *L*. *salivarius* strains was tested, and significant differences were observed between the groups (Fig 6A). SAPs formed more biofilm than SBPs after 48 and 72 h of cultivation. SAPs formed 11-fold more biofilm in 48 h and 30-fold in 72 h than SBPs. Quantification of dye bound to biofilm for SBPs showed 3.79 ± 1.62 μg in 48 h and 2.05 ± 0.57 μg in 72 h, while SAPs showed 40.25 ± 4.64 μg in 48 h and 62.38 ± 4.72 μg in 72 h.

The strains that form more biofilm than others have a survival advantage on the competing species and can become a dominant bacterium in certain habitat, such as the porcine intestine. We corroborated the survival rate of SAPs in competition with swine intestinal microbes (Fig 6B and S3 Fig). The number of SAP cells increased after 48 h compared to 24 h, while the SBP cells had decreased. The number of *L*. *salivarius* cells in co-cultures of SAPs and intestinal microorganisms for 24 h did not differ from SBPs. However, after 48 h of competition, the number of SAP cells was higher than SBPs (P = 8.922 × 10^−10^). These results indicate that SAPs were suitable to survive because of their ability to form biofilm.

## Discussion

We isolated the *L*. *salivarius* strains at an interval of a decade, and the use of AGPs was legally banned in Republic of Korea during this period. SBPs and SAPs were isolated from the two time points, before and after the ban on AGPs, respectively. The ban on using AGPs changed the conditions of the livestock industry, including the physiological characteristics of the farm animals and their commensal bacteria. It is well documented that alpha diversity of the intestinal microbiota was decreased following antibiotic perturbation in animals [5–7]. Our investigation revealed the bacterial genomic dynamics affected by the AGP prohibition. After the prohibition, the intestinal environment colonized by gut microbiota was altered, and the intestinal bacteria evolved for adaptation to the changed habitat. Although many researchers showed that genomes of microorganisms are influenced by antibiotic selection pressure [11, 25, 26], there is limited evidence on the effect of antibiotic removal on the bacterial genome.

This study showed that the evolutionary distance among SBPs was more adjacent than SAPs, although SBPs were isolated from more numerous farms than SAPs. Six SBPs were isolated from five farms and fifteen SAPs from three farms (Fig 1 and Table 1). The genomic features of SBPs, including the number and the contents of orthologs, were barely different among the six SBPs. However, the genomic profiles of SAPs were more diverse than SBPs. This result may explain two evolutionary points: 1) AGPs in the feeds had caused the unremitting and strong selective antibiotic challenge to intestinal bacteria; 2) After the prohibition of AGPs, intestinal bacteria had been released from the antibiotic selection pressure, and this situation allows more evolutionary freedom. The ban of AGPs forced ancestral strains of SAPs to gradually change their genomic contents through the movement of genetic material (e.g., horizontal gene transfer). During the evolutionary process, ancestral strains of SAPs would have discarded several genes that were not needed for the survival of the bacteria, leading to the smaller genome size and decreased number of CDSs of SAPs compared to SBPs.

All of the antibiotics targeted by AR genes of SBPs and SAPs have been used as AGPs in swine farms of Far-East Asia [27, 28], and genes that target the antibiotics described above were found in other bacterial species isolated from the feces of pigs and cattle in Republic of Korea [29]. Interestingly, a number of AR genes in the *L*. *salivarius* genomes were retained after AGP prohibition, although other genomic characteristics, such as genome size and amino acid sequence of biofilm formation-associated genes, were changed for adaptation to the environment that antibiotics are not ever-present. It is assumed that SAPs have no reason to discard AR genes after AGP prohibition. Antibiotics have been still used in animals for a veterinary purpose, and this situation made the bacteria keep AR genes in their genomes.

Swine intestinal microbes that did not have AR genes could not protect themselves from AGPs when antibiotics were added to animal feeds. Furthermore, several studies showed that intake of antibiotics decreased the diversity of microbiota in the animal intestines [5, 6], although the total number of intestinal bacteria was not changed [7]. In these circumstances, the competition between microbes for survival and domination in the porcine intestines would be decreased, and bacteria that had AR genes are more advantaged for survival in this microenvironment. On the other hand, increased competition after AGP prohibition required another strategy for survival among bacteria. They had to evolve for overcoming this severe struggle, and they improved their ability for biofilm formation. It is well documented that biofilm provides the sufficient power to oppose other microbes [30, 31]. Bacteria isolated from the time when AGPs were added in the feeds did not need to form biofilm because AGPs decreased the competition among microbes. Indeed, SBPs barely formed any biofilm compared to SAPs. We observed that genes associated with biofilm formation were different between the groups at the amino acid sequence level. EPSs and extracellular proteins are important components of *Lactobacillus* cell wall and biofilm [21, 23]. This suggests that the differences in these genes would have a relationship with the amount of biofilm produced by SBPs and SAPs, and dense biofilm formation leads to increased survivability of SAPs from competition with intestinal microbes.

The presence of genes participating to the metabolic pathways of energy production would affect the sparse biofilm formation of SBPs. Compared to SAPs, SBPs have additional genes for energy production, including L-rhamnose utilization and D-tagatose and galactitol metabolism. L-rhamnose is the main structural component of EPSs produced in *Lactobacillus* spp. [18, 19], and D-tagatose also contributes to EPS synthesis and lactic acid fermentation for energy production in LAB [20]. It is possible that SBPs use L-rhamnose as an energy source rather than for EPS production, and the genes for D-tagatose utilization are also associated with this energy production process. Due to the additional genes for sugar utilization, SBPs have more available energy than SAPs, and this energy would be used for other biological processes, such as choline and betaine uptake and betaine biosynthesis, and DNA replication strays (Fig 3). Moreover, SBPs showed a broader genetic capacity than SAPs, such as genome size and number of orthologs, and 335 unique orthologs appeared in the SBP genomes, although SAPs has 103 unique orthologs. These differences in the genetic profiles suggest that the extra energy of SBPs would be used for other processes rather than EPS production.

The ability for biofilm formation that helps to survive in the competition with intestinal microorganisms is an important probiotic property. *L*. *salivarius* has many features as probiotic, including antimicrobial activity against pathogenic bacteria [32, 33], reduction of pathogen adhesion to surfaces [34], bile salt hydrolase activity [35], indirect support of energy metabolisms by microbial fermentation [36] and effects on host immunocompetent cells [37]. To maximize these probiotic functions, *L*. *salivarius* have to colonize and survive in the gastrointestinal tract, and biofilm formation helps this process. This spatial organization of microbes attached to the intestinal surfaces subdivides the role of the members and protects themselves from harmful substances and other competitive microorganisms [30, 38, 39].

To the best of our knowledge, this is the first report on the effect of AGP prohibition on the changes in genomic features of animal intestinal bacterium based on genome sequencing. Although more studies are required to understand the relationship between biofilm formation and the genomic profile of *L*. *salivarius*, our study provides evidence that the ban of AGPs, which had been commonly added in feeds for growth promotion of livestock, can modify the bacterial genome to adapt to the altered microenvironment. This study also suggested that the change in the genomic profiles occurred not only in *L*. *salivarius* but also other bacteria. Furthermore, given the many functional properties of *L*. *salivarius* as probiotic, the genetic features allowing dense biofilm formation can provide detailed insight for screening and selection of probiotic *L*. *salivarius* strains, and additional in-depth research examining these genetic features would be worthy of investigation.

## Materials and methods

### Preparation of *L*. *salivarius* genomes and ortholog identification

We had previously reported isolation, identification, and genome sequencing of the *L*. *salivarius* strains used in this study [16]. ANIb, indicative of the relationships among species, was calculated by JSpeciesWS [40]. All of the genome sequences of *L*. *salivarius* strains obtained are deposited into NCBI Whole Genome Shotgun (WGS) database (Table 1).

All CDSs of the draft genomes were collected and orthologs were identified as previously described [41]. Briefly, CDSs in the annotations were filtered to remove CDSs containing premature stop codons (pseudogenes). Each CDS was then aligned to the entire CDS pool using GASSST, according to nucleotide sequence identity (≥ 85%) and best sensitivity [42]. The aligned CDSs were regarded as one ortholog, and the consensus sequence of each ortholog was determined using the CAP3 with default options [43].

### Functional comparison of CDSs including AR, EPS and extracellular protein genes

The assembled draft genome sequences were uploaded to RAST server with default options for bacteria to obtain information on gene functional categories, called subsystems [44]; and the gene amounts were counted for each subsystem.

Cell wall-anchored proteins with a choline-binding domain (Pfam family: CW_binding), LPXTG domain (Gram_pos_anchor), lipoprotein anchor (Lipoprotein_Ltp), LysM domain (LysM), peptidoglycan-binding domain (PG_binding) and WXL domain (WXL) were identified for further analysis using the Pfam database [45]. For analyses of genes associated with EPS production, 50 EPS-related genes of *L*. *salivarius* UCC118 were collected from a previous study [21], and the pan-genome orthologs that belonged to the same Pfam family of EPS genes in UCC118 were obtained and filtered manually. Sequences of AR genes were obtained from the CARD [46]. Orthologous CDSs of the *L*. *salivarius* genomes were aligned to AR genes using MUSCLE for detection of the genes in each genome [47]. Sequence identity was calculated by BLAST+ [48] and each ortholog having E-value = 0 and identity ≥ 90% was regarded as the identical gene in the list of the analyzed genes.

### Hierarchical clustering of the genomes

The presence or absence of each orthologous CDS in a genome was used for hierarchical clustering using the Euclidean distance method implemented in the R [49]. Existence of orthologs was statistically examined by 1,000 bootstrapping using an R package, Pvclust [50].

Phylogenetic analyses based on nucleotide or amino acid sequences were carried out using MEGA7 [51]. The nucleotide or amino acid sequences were retrieved through the global alignment of orthologous CDSs from each genome and were compared using the multiple sequence alignment software MUSCLE 3.8.31 [47]. The phylogenetic relationship was inferred using the Neighbor-Joining method [52]. The bootstrap consensus tree inferred from 1,000 replicates was taken to represent the evolutionary history of the taxa being analyzed [53]. The evolutionary distances were computed using the Maximum Composite Likelihood method [54]. Nucleotide sequences of the seven *L*. *salivarius* housekeeping genes, *pstB*, *rpsB*, *pheS*, *ftsQ*, *nrdB*, *rpoA* and *parB* were used in clustering for MLSA [21].

### *In vitro* biofilm formation assay

Biofilm formation of *L*. *salivarius* strains was analyzed as described by P. Ambalam *et al*. [55] with some modification. Each well of sterile TPP flat-bottomed 96 well microplates was filled with 200 μl of MRS broth. Overall, ~2 × 10^6^ *L*. *salivarius* cells were added to each well and incubated under static conditions at 37°C for 48 and 72 h. The plates were then washed twice with PBS and dried for fixation at 55°C for 20 min. All plates were washed three times with phosphate-buffered saline (PBS) and the bacteria attached to the surface were stained with 200 μl of 0.1% (w/v) crystal violet in 1: 1: 18 of isopropanol-methanol-PBS solution (v/v). Excess dye was rinsed off by washing three times with PBS. The residual dye bound to the surface-adhered cells was extracted with 200 μl of 30% glacial acetic acid, and the absorbance of each well was measured at 630 nm in a microplate reader (Infinite M200 Pro, Tecan, Zürich, Switzerland). The amount of surface-bound dye was determined using a standard curve for crystal violet (μg).

### *In vitro* competition assay with swine fecal microorganisms

Fecal microbes were prepared according to a previous study with some modifications [56]. Feces were obtained from eight pigs that did not take any probiotics or antibiotics, and two grams of the mixed feces were placed in 30 ml of pre-reduced PBS. The fecal material was suspended by vortexing for 5 min, and the suspension was allowed to stand at room temperature for 10 min. Supernatant of the suspended feces sample was diluted, and ~5 × 10^7^ fecal microbes were plated on each well of 24-well plates containing 1.6 ml of pre-reduced brain-heart infusion (BHI) broth (BD, NJ, USA). *L*. *salivarius* strains were cultured in MRS broth for 24 h at 37°C and the cells were washed with PBS. 1 × 10^5^ cells of each strain were added to each well of the fecal microbe-containing plates. They were incubated at 37°C for 24 and 48 h, and then the cells were harvested for *L*. *salivarius* quantification. All of the processes were carried out under anaerobic condition (an atmosphere of 75% N_2_, 20% CO_2_ and 5% H_2_).

The amount of *L*. *salivarius* strains was calculated by real-time PCR with *L*. *salivarius*-specific primers, For-Sal-3 (5’-GTCGTAACAAGGTAGCCGTAGGA-3’) and Rev-Sal-1 (5’-TAAACAAAGTATTCGATAAATGTACAGGTT-3’) [57]. gDNA of the strains was extracted by microwaving as described above. Amplification reaction mixtures (total volume 20 μl) contained 40 ng of the extracted gDNA, 10 μl of SYBR green qPCR 2X premix (Enzynomics, Daejeon, Korea) and 500 nM of each primer. Tests were performed with CFX96 Touch Real-Time PCR Detection System (BioRad, CA, USA). PCR was performed as follows: 50°C for 2 min, 95°C for 10 min, 40 cycles of 95°C for 15 sec and 63°C for 1 min. To determine the specificity of the SYBR green PCR assay, a melting curve analysis of the DNA fragments was performed according to the manufacturer’s instructions. Standards were used to determine the amount of *L*. *salivarius* DNA by real-time PCR using a modified protocol of a previous study [58]. Purified gDNA ranging from 1 pg to 100 ng of *L*. *salivarius* KLF003 was used as the standard. This was equivalent to approximately 4.16 × 10^2^ to 4.16 × 10^7^ copies of the genome (the average genome size of the strains used in this study is 2.21 Mbp).

### Statistical analysis

We used Student’s t-test for the comparison of genomic features, *in vitro* biofilm formation assay and *in vitro* competition assay between SBPs and SAPs [59]. In the comparison of RAST subsystems, differentially over-represented gene numbers in each subsystem were examined between SBP and SAP genomes using Fisher’s exact test [60]. The statistical significance in all analyses was set at P < 0.05.

## Supporting information

S1 Fig Distribution of orthologous CDSs in the 21 *L. salivarius* strains isolated in this study. Distribution histograms are shown for the 21 *L*. *salivarius* strains. The horizontal axis indicates the number of isolates sharing the same orthologous CDSs, and vertical axis represents the number of orthologous CDSs shared by the indicated number of isolates.
(TIF)

S2 Fig Difference in cell aggregation of SBPs and SAPs. For this test, the *L*. *salivarius* strains were cultured for 24 h at 37°C in shaking condition (240 rpm). Cell aggregation of the cultured bacteria was observed after incubation for 2 h at 37°C in static condition. NC, negative control (MRS broth only).
(TIF)

S3 Fig Competition assay with swine intestinal microbiota. Purified gDNA of *L*. *salivarius* KLF003 was used as the standard to calculate the cell numbers (see Materials and Methods).
(TIF)
