# Supplementary figures and images for: Prohibition of antibiotic growth promoters has affected the genomic profiles of Lactobacillus salivarius inhabiting the swine intestine
Source: PLoS One. 2017 Oct 23;12(10):e0186671. doi: 10.1371/journal.pone.0186671 (PMC5653324; doi:10.1371/journal.pone.0186671)

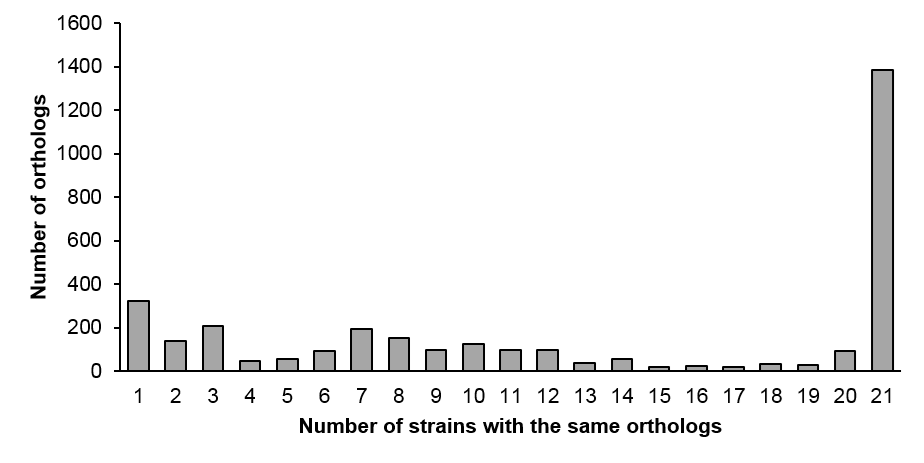

Supplement: S1 Fig — Distribution histograms are shown for the 21 L. salivarius strains. The horizontal axis indicates the number of isolates sharing the same orthologous CDSs, and vertical axis represents the number of orthologous CDSs shared by the indicated number of isolates. (TIF) [file pone.0186671.s001.tif]

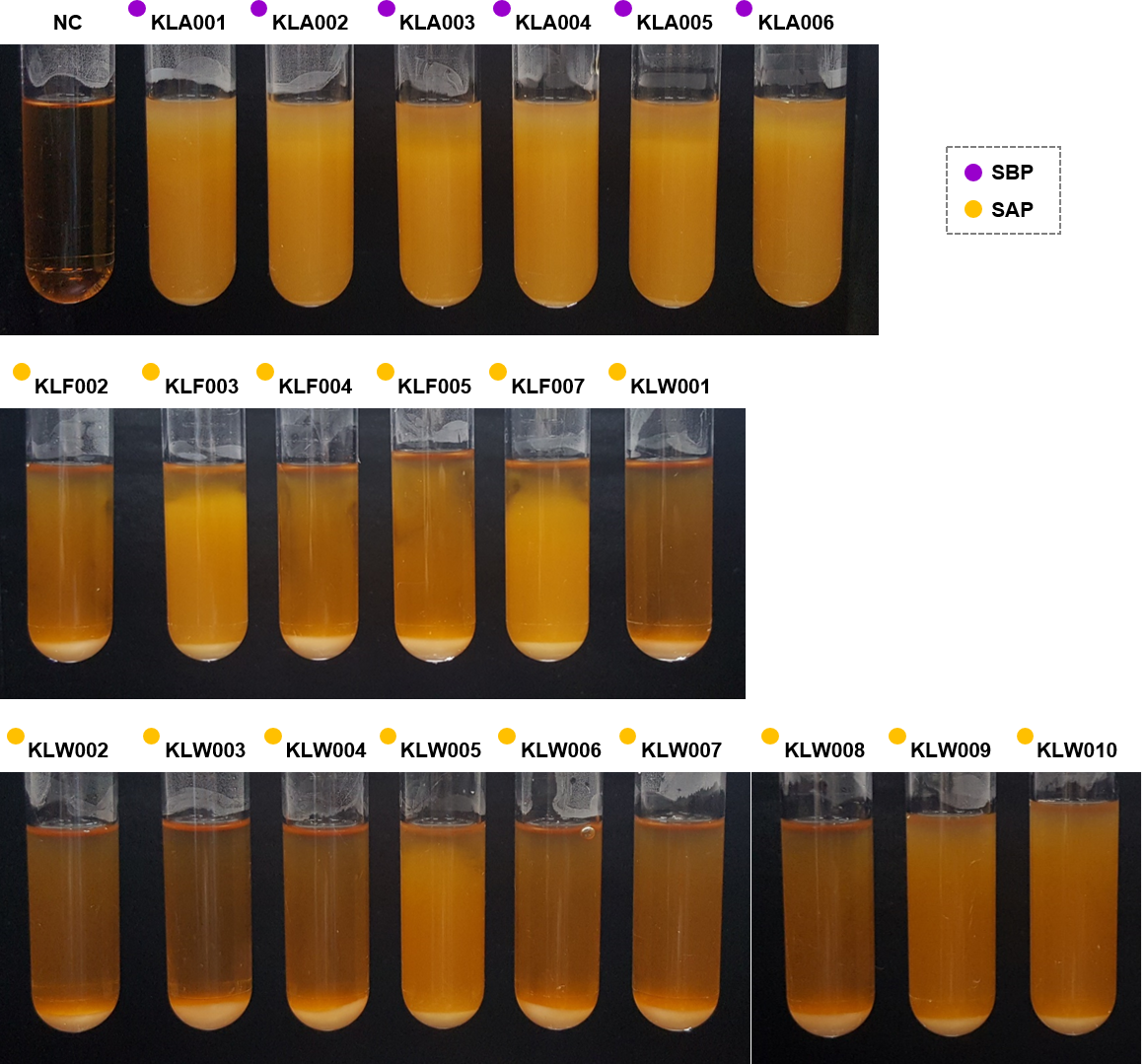

Supplement: S2 Fig — For this test, the L. salivarius strains were cultured for 24 h at 37°C in shaking condition (240 rpm). Cell aggregation of the cultured bacteria was observed after incubation for 2 h at 37°C in static condition. NC, negative control (MRS broth only). (TIF) [file pone.0186671.s002.tif]

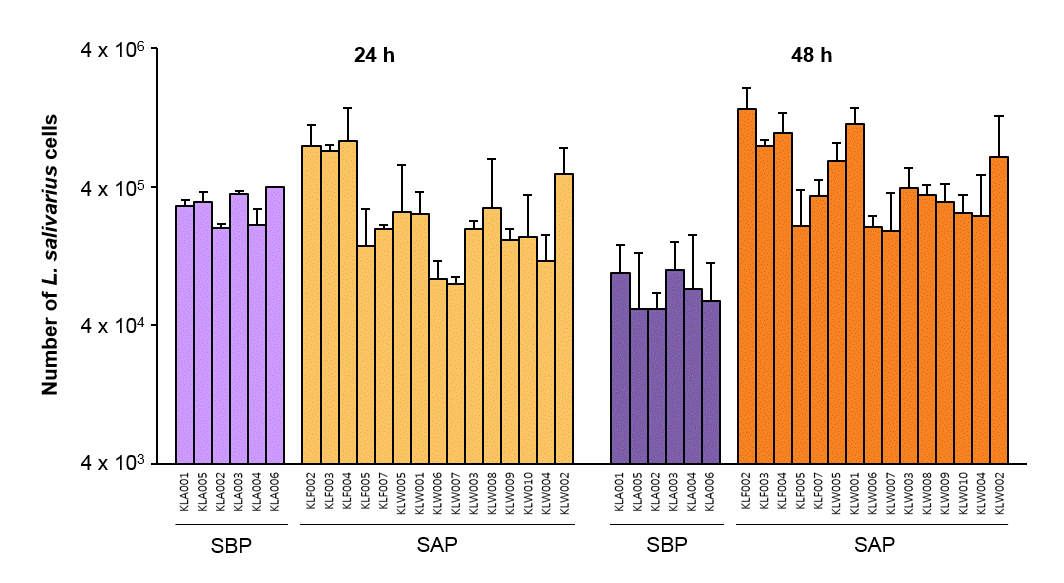

Supplement: S3 Fig — Purified gDNA of L. salivarius KLF003 was used as the standard to calculate the cell numbers (see Materials and Methods). (TIF) [file pone.0186671.s003.tif]
